# Supplementary figures and images for: Evolution of basic human values orientations: An application of monitoring changes in cluster solutions
Source: PLoS One. 2022 Sep 30;17(9):e0274600. doi: 10.1371/journal.pone.0274600 (PMC9524711; doi:10.1371/journal.pone.0274600)

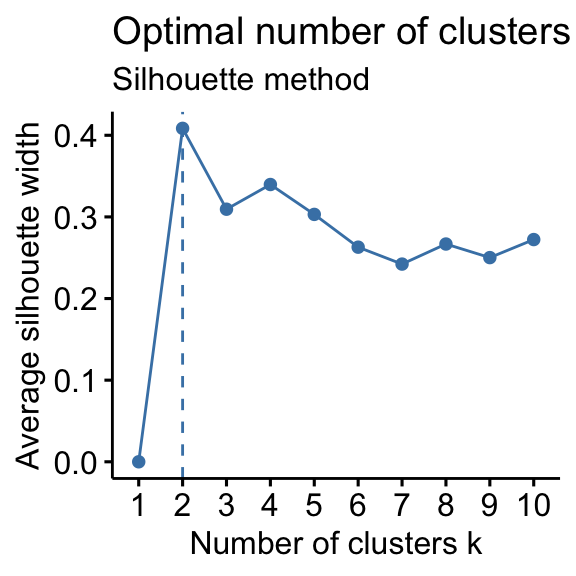

Supplement: S1 Fig — These packages provides different options, which also includes the average silhouette statistics. The average silhouette method computes the average silhouette of observations for different values of k. The optimal number of clusters k is the one that maximizes the average silhouette over a range of possible values for k. For example in the Figure below there are k = 2 natural subgroups or clusters in the dataset. (PNG) [file pone.0274600.s001.png]
