# Supplementary material for: Evolution of basic human values orientations: An application of monitoring changes in cluster solutions
Source: PLoS One. 2022 Sep 30;17(9):e0274600. doi: 10.1371/journal.pone.0274600 (PMC9524711; doi:10.1371/journal.pone.0274600)
Supplement: S1 Appendix — (PDF) [file pone.0274600.s002.pdf]

Table 4. Items in SVS questionnaire and their associated human values.

| Human Value | Number | Item |
|---|---|---|
| BENEVOLENCE | 12 | It's very important to him to help the people around him. He wants to care for other people. |
| | 18 | It is important to him to be loyal to his friends. He wants to devote himself to people close to him. |
| UNIVERSALISM | 03 | He thinks it is important that every person in the world be treated equally. He wants justice for everybody, even for people he doesn't know. |
| | 08 | It is important to him to listen to people who are different from him. Even when he disagrees with them, he still wants to understand them. |
| | 19 | He strongly believes that people should care for nature. Looking after the environment is important to him. |
| SELF-DIRECTION | 1 | Thinking up new ideas and being creative is important to him. He likes to do things in his own original way. |
| | 11 | It is important to him to make his own decisions about what he does. He likes to be free to plan and to choose his activities for himself. |
| STIMULATION | 06 | He likes surprises and is always looking for new things to do. He thinks it is important to do lots of different things in life. |
| | 15 | He looks for adventures and likes to take risks. He wants to have an exciting life. |
| HEDONISM | 10 | Having a good time is important to him. He likes to "spoil" himself. |
| | 21 | He seeks every chance he can to have fun. It is important to him to do things that give him pleasure. |
| ACHIEVEMENT | 04 | It is very important to him to show his abilities. He wants people to admire what he does. |
| | 13 | Being very successful is important to him. He likes to impress other people. |
| POWER | 02 | It is important to him to be rich. He wants to have a lot of money and expensive things. |
| | 17 | It is important to him to be in charge and tell others what to do. He wants people to do what he says. |
| SECURITY | 05 | It is important to him to live in secure surroundings. He avoids anything that might endanger his safety. |
| | 14 | It is very important to him that his country be safe from threats from within and without. He is concerned that social order be protected. |
| CONFORMITY | 07 | He believes that people should do what they're told. He thinks people should follow rules at all times, even when no-one is watching. |
| | 16 | It is important to him always to behave properly. He wants to avoid doing anything people would say is wrong. |
| TRADITION | 09 | He thinks it's important not to ask for more than what you have. He believes that people should be satisfied with what they have. |
| | 20 | Religious belief is important to him. He tries hard to do what his religion requires. |
