# Supplementary material for: Evolution of basic human values orientations: An application of monitoring changes in cluster solutions
Source: PLoS One. 2022 Sep 30;17(9):e0274600. doi: 10.1371/journal.pone.0274600 (PMC9524711; doi:10.1371/journal.pone.0274600)
Supplement: Attachment — Submitted filename: Response to Reviewers.docx [file pone.0274600.s003.docx]

**Reviewer 1:**

- **Introduction:**
- The short introduction is well structured with the main message that changing basic human values influence human actions and changes therein. But the goal of the study is to "to investigate the dynamics of the relationship between" basic human values. I miss a short theoretical reflection about these value changements.

**Response: A paragraph explaining the theory of dynamic relationships amongst the human values is added to the introductions section.**

- At the end of the introduction, the authors write that they want "to achieve the aforementioned objectives". But explicitly, they mention only one goal.

**Response: A subsection “Research objectives” is added to the manuscript to clearly explain the main aims and objectives of the study.**

- The literature (of the paper) is not very up-to-date. Apart from technical references (e.g. on R), the references are all before 2014 with one exception (2018). For example, the last paragraph on page 2 begins with "In recent years, one of the most exciting topics is the study of diversity and cultural changes over time." The following sentences are taken from Davidov (2008). That is, by in recent years, the authors are referring to a paper written 14 years ago. Or "Yet recent development" refers to 1992 and 2002. In fact, there is not much literature on "basic human values" and on "Schwartz Value Survey". But there are recent studies on the topic. The theoretical background should therefore be updated.

**Response: More up-to-data references are added to the article.**

- **Methodology**
- Chapter 2 states a slightly different aim of the study: "in order to recognize any variation in value score over time and its impacts on the cultural groups".

**Response: We added a new subsection “Research objectives” at the end of “Introduction” to clarify and elaborate the objectives of this research article.**

- Why do the authors quote Eurostat (2011) when there is new data from 2021 (immigrants in Europe: 5.1% instead of 4.4%)?

**Response: The reference is updated.**

- Chapter 2.1: Here I miss the number of participants in the eight surveys or at least a reference to Table 1 and some more information about the samples and the data collection.

**Response: Table 1 represents the cumulative number of participants in these surveys. The second row in the Table represents the number of participants.**

- Is there a literature reference for choosing the number of clusters? <- "The choice of an optimal number of clusters was decided by silhouette width, a measure of internal validity. Multidimensional scaling was used for the graphical representation of relation amongst human values in important clusters."

**Response: Details on silhouette measure is added to the manuscript. Details on the output of R packages factoextra() and Nbclust() is added in the supplementary information to clarify the use of silhouette measure. A detail on the multidimensional scaling is also added to the manuscript. The MDS was used to examine the relationship between values and identify the similar values that are emerging.**

- The procedure of the analysis has been described in an understandable and comprehensible way. It would be helpful if the authors indicated in the methodology section how the quantity *w* is obtained.

**Response: There is no method for estimating the width of sliding window in the sliding window model. However, as declared in the manuscript if we use landmark window or sliding window with high value of w then the cluster are effected by out dated historical observations. That is the reason we used only resent observations for generating the cluster solutions.**

- The method of multidimensional scaling is not reported.

**Response: Details on multidimensional scaling is added to the manuscript.**

- **Results and discussion**
- I would rename this section in "Results"

**Response: We added discussion on each table and figure in the result section and therefore keep the sections as “Results and discussion”.**

- It would be beneficial for the reader if Figure 1 were described in more detail. Figure 1 contains four illustrations. The text does not explain which figure shows what.

**Response: Details are added to the Figure 1.**

- Why do the authors not report the density measures?

**Response: The density was measured by the size of each cluster, which is given in the last row of the Tables for values scores.**

- Figures 3 and 4 are not self-speaking and should therefore be better explained in the text. It would also be helpful to write out the scales of the basic human values in the figure instead of using abbreviations.

**Response: Explanation is added to the Figures 3 and 4.**

- **Summary and conclusion**
- The sentence "Mostly the values change studies based on determining the directions in which people’s actions, thinking and feelings have endured transformation." needs some references (or I do not understand it).

**Response: To clarify the text, it is rephrases and explanation is added to the manuscript.**

- This discussion correctly summarises briefly what was found in the analyses. In the last section I can see a conclusion.
  The results (content) are not discussed or linked to theoretical findings and previous studies. I also miss a discussion of the significance of the results. What does it mean for a society to give more space to universalism? How can this be reconciled with the world situation today (especially in Europe)?

**Response: Added to the conclusion seciton in revised manuscript.**

- I also miss a discussion on alternative explanations (content-related, methodical). The article does not give any statistical data on the Schwarz Value Survey. The scales each consist of two questions. The fact that there are only two stable clusters could also be due to a lack of reliability or other problems with the scales. Or how stable is the construct of basic human values over time? How stable are human basic values over time?

**Response: The data on Schwartz Value Survey are openly available from the European Social Survey website homepage. Usually the values are consistent and the change is very slow. The reason for such a large number of disappearing clusters was the migrants. The migrants changes values very rapidly which also effects the culture of the host nations.**

- Furthermore, the weaknesses and strengths of the study are not addressed.

**Response: Addedto the manuscript**

**Reviewer 2:**

- The article begins with the Introduction section. I understand it should be as short as possible, but the concept of value should be developed. What are the values and how are they understood (definition). Schwartz defines individual values in relation to the goals that motivate actors to act and guide that action. Values are also the source of standards and are ranked in order of importance. Of course, Schwartz's theory is often used, but I'm not sure it's the most popular.

**Response: The Theory of Basic Human Values is a theory of cross-cultural psychology and universal values that was developed by Shalom H. Schwartz. The theory extends previous cross-cultural communication frameworks such as Hofstede's cultural dimensions theory. Furthermore, the Schwartz theory is used because it also explains the relationship between the basic values. For example the pursuit of one value may affect the pursuit of other values. For this reason we used the SVS.**

- While the statistical part is described quite accurately, and I have no comments (besides some limitations in using data from ESS) in the section: result and discussions there is no discussion. What is the answer to your main questions? What are the consequences? What do the results explain?

**Response: The discussion on the Figures and Tables are added to the revised manuscript. The results and main findings of the study are elaborated.**

- Even thought in the last part: summing up and concluding some ideas about universalism and living anxiety-free life are not enough. I would appreciate elaborating these ideas. What does it mean? And what are the roots of these changes and what are the consequences? What are the differences in Europe? What about migrants?

**Response: Discussion on the tables and figures are added to the manuscript. And the meanings of emerging values are elaborated in the revised manuscript.**
