# Supplementary material for: Evolution of basic human values orientations: An application of monitoring changes in cluster solutions
Source: PLoS One. 2022 Sep 30;17(9):e0274600. doi: 10.1371/journal.pone.0274600 (PMC9524711; doi:10.1371/journal.pone.0274600)
Supplement: Attachment — Submitted filename: Response to Reviewers.docx [file pone.0274600.s004.docx]

### Response to Reviewers

### Thank you for giving me the opportunity to submit a revised draft of my manuscript titled *Evolution of Basic Human Values Orientations: An Application of Monitoring Changes in Cluster Solutions* to PLOS ONE. We appreciate the time and effort that you and the reviewers have dedicated to providing your valuable feedback on our manuscript. We are grateful to the reviewers for their insightful comments on my paper.

Here is a point-by-point response to the reviewers’ comments and concerns.

A) The following comment was not included in your response-to-reviewers: "My main concern is Europe - Europe seems to be treated as one 'site', while Europe is very diverse in terms of value, but also in terms of the migration that you mentioned. I would also add the perspective of transformation in the countries of Eastern Europe. Perhaps Hofstede's theory could help investigate this."

**Response: This is added to the manuscript as limitations of the study.**

B) The reference to Eurostat was updated in the tracked changes version of the manuscript, but not the clean copy. Please ensure the content of the two documents is identical.

**Response: The reference in the clean copy and track changes copy is updated, but it was not highlighted in the track changes copy. It is highlighted in track changes copy.**
